# Breakdown products of the fungicide Fludioxonil may account for observed environmental impact: potential implications for human health

**DOI:** 10.7717/peerj.21290

**Published:** 2026-06-03

**Authors:** Laura Roelans, Tristan Brandhorst, Marco Tonelli, Grazia Chiellini, Warren P. Porter

**Affiliations:** 1 Department of Integrative Biology, University of Wisconsin-Madison, Madison, WI, United States of America; 2 Department of Biochemistry NMRFAM, University of Wisconsin-Madison, Madison, WI, United States of America; 3 Department of Surgical, Medical and Molecular Pathology and Critical Care Medicine, University of Pisa, Pisa, Italy

**Keywords:** Fludioxonil, Fungicide, Glutathione, Toxicity, PFAS, Environmental health

## Abstract

**Background:**

Fludioxonil fungicide has been shown to rapidly degrade in the environment into a perfluoroalkyl substance (PFAS) breakdown product. Fludioxonil has been previously associated with both health and environmental damage, but a clear mechanism has not been proven and reports of toxic effect have been troublingly inconsistent.

**Methodology:**

This review collects references from 2012–2025 detailing the extent of ecological impact and human health effects ascribed to fludioxonil. An effort was made to focus on points of similarity to shed light on potential mechanisms.

**Results:**

It is found that prior observations of toxic effect may be explicated by an elicitation of oxidative stress. We illustrate here that a primary breakdown product of fludioxonil includes a maleimide moiety, a chemical structure capable of irreversibly binding biological thiols like glutathione (GSH). This is relevant because damaging this key component of cellular anti-oxidant defense is known to be causal for increased oxidative stress.

**Conclusions:**

A mechanism of toxicity based on GSH depletion is consistent with previously unexplained toxicity issues ascribed to this fungicide, emphasizing the potential threat to both agriculture-adjacent aquatic biomes (from fish to phytoplankton) and human health.

## Concerns Regarding Fludioxonil Safety: Properties and Ecological Effects

### Introduction

**Figure 1:**
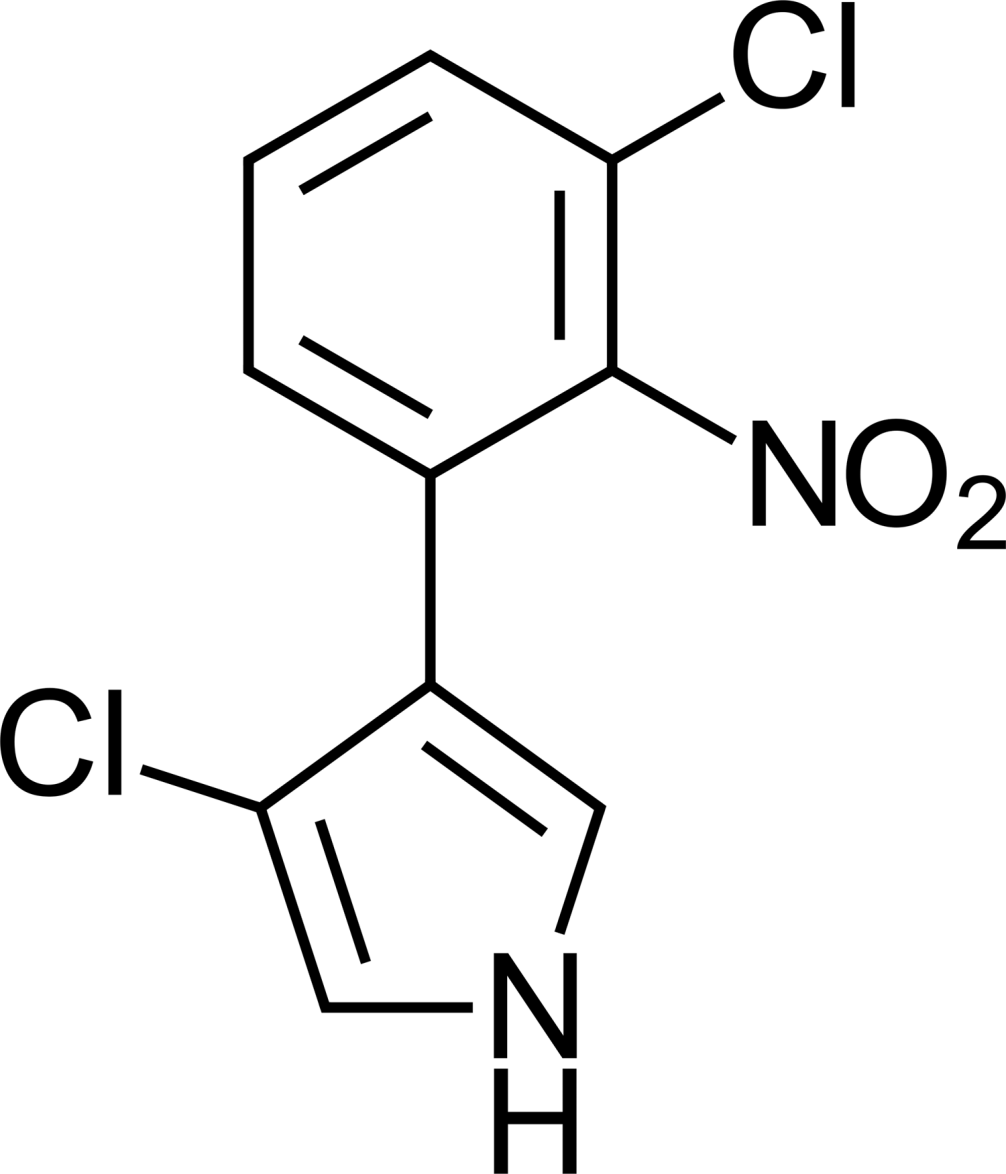
Pyrrolnitrin toxin produced by *Pseudomonas* species.

**Figure 2:**
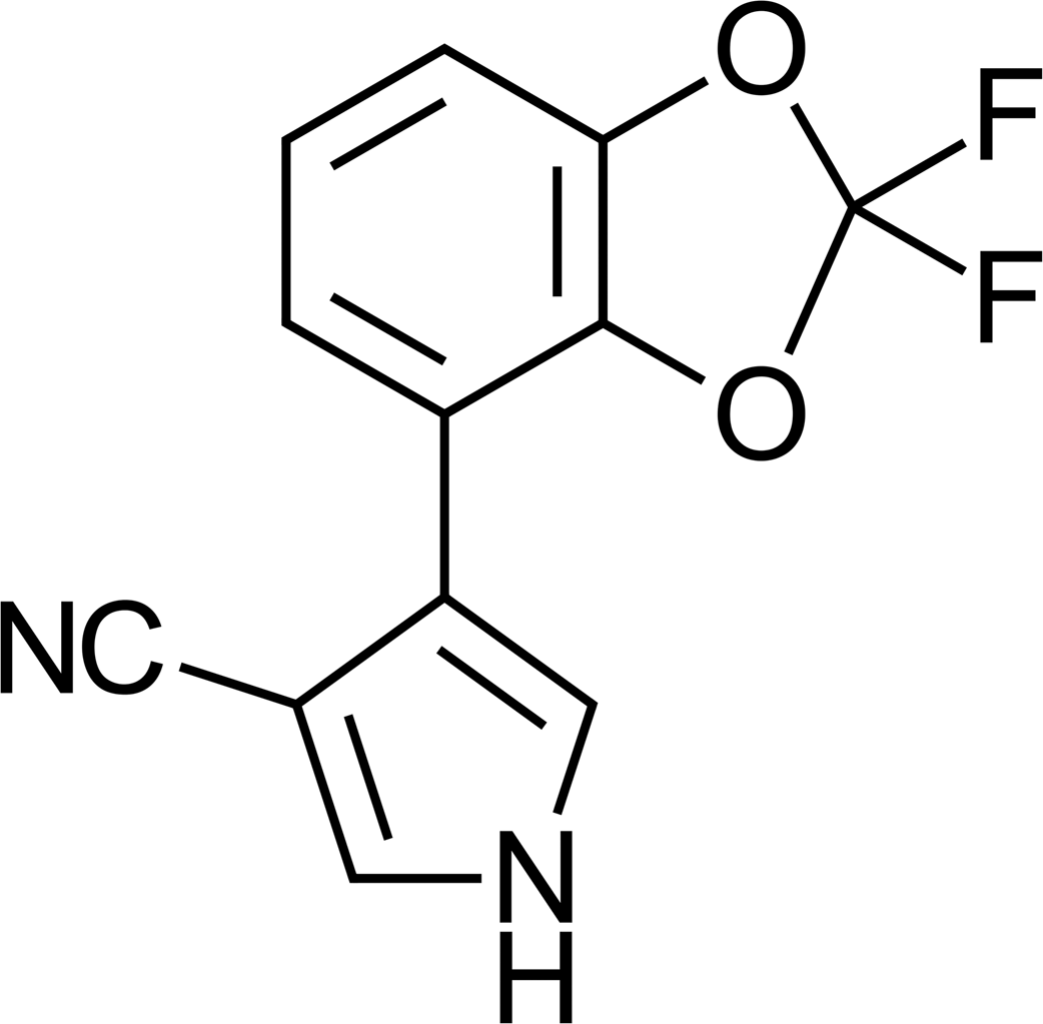
Fludioxonil fungicide.

The fungicide fludioxonil, developed by the agrochemical company Syngenta (Ciba Geigy at the time) in 1993, was derived from the bacterial toxin pyrrolnitrin (Fig. 1) which was derived from *Pseudomonas* species. Fludioxonil (Fig. 2) was originally engineered from pyrrolnitrin to create a preservative/anti-fungal coating for seed storage. Products including fludioxonil performed reliably and effectively as preservative and anti-fungal agents. When Syngenta sought to register fludioxonil as a multi-use fungicide with the US EPA the company presented data indicating that fludioxonil functioned by acting directly upon a single enzyme, a kinase that was unique to specific species of fungi (Kojima et al., 2004). This proposed mechanism of action, as described, may have biased regulators to anticipate that fludioxonil would be harmless to non-target organisms while remaining deadly to the fungi it was designed to kill.

Fludioxonil was marketed to curtail mold spoilage while promising safety for crops, consumers and the environment. In disclosures to the US EPA, fludioxonil was described as being resistant to ultraviolet (UV) photolysis/hydrolysis and free from concerns regarding toxic synergy with other pesticides. With these assurances, it is not surprising that fludioxonil use increased and was adapted to myriad applications, enhancing its usage in new markets worldwide. In the United States alone this fungicide is registered for use on over 900 different types of produce. A French study found that fludioxonil was among the seven pesticides most likely to reach consumers through residual presence in food (Graillot et al., 2012) and is frequently found in imported produce, often at levels that surpass regulatory limits, such as produce exported from Turkey and Argentina (Gormez et al., 2025) where it is the primary fungicide in use.

Uses of fludioxonil now include large-scale application by sprayer to crops to prevent fungal infections during growth, but also include post-harvest applications, often *via* drench/drip or spray to prevent mold during transit to grocery stores. The highly hydrophobic fungicide is particularly difficult to wash off of produce, ensuring that a substantial percentage of the fungicide used in treatment remains on food at time of consumption.

Heavy usage as a post-harvest mold retardant places fludioxonil in a class by itself. Though fludioxonil is often added to produce after it has been boxed for shipment to market, serving as a preservative, the US FDA, per their last review of available science, did not choose to categorize fludioxonil as a food additive. Consequently, regulation of fludioxonil lies under the jurisdiction of the US EPA alone, which does not distinguish this form of concentrated application from pesticides that may take days or even weeks to be degraded in the field, detoxified or leached from the surface of produce by natural actions of sunlight, wind, rain and metabolism by microorganisms. This situation can result in high levels of this pesticide reaching and being ingested by consumers- for example, an Environmental Working Group survey of pesticide levels in common brands of baby food found fludioxonil significantly exceeded the Maximum Residue Level (MRL) established by the US EPA as recently as 2018 (Oluwaseun & Tawakalitu, 2018).

In contrast to initial reports indicating fludioxonil was non-toxic to non-fungal targets, evidence has emerged that fludioxonil exerts adverse effects upon a wide array of organisms besides fungi (Lawry et al., 2017; Brandhorst & Klein, 2019). In particular, aquatic organisms seem to be susceptible to its action (Wang et al., 2020), but the list also includes plants (Liu et al., 2022), bacteria (Lassalle et al., 2015), nematodes (Choi et al., 2025), benthic macroinvertebrates (Sun et al., 2022), human lymphocytes (Antonopoulou et al., 2025) and terrestrial earthworms (Haegerbaeumer et al., 2019). Given this broad spectrum of toxicity, it may be of concern that this fungicide is widely applied to many of the staple foods consumers ingest regularly.

In this review, we will present the accumulated evidence that fludioxonil poses a threat to both ecosystems and human health through a recently established capacity to induce oxidative stress through exhaustion of protective glutathione (GSH), this being driven by the reactive capacity of a maleimide moiety which is rapidly formed during the breakdown of fludioxonil by the UV radiation present in sunlight.

### Methodology

This literature review is intended for researchers, both corporate and academic, risk managers, developers of governmental policy, and concerned citizens with an interest in safe agricultural practices and food hygiene. We performed a search of peer-reviewed articles, patents, policy documents, and preprints published between 2012 and 2025 to outline the weight of evidence supporting the premise presented here. Search engines used included PubMed and the NIH rePORTER, due to their extensive coverage of high-quality, peer-reviewed biomedical and clinical research literature, but Google was used as well to discover relevant information that may not have been indexed in more specialized databases. It is recognized that preprints are of potential interest in this field, so Google Scholar was also incorporated into our searches. Databases inspected directly included Govinfo for federal governmental minutes, USPTO Patent Public Search for patent information, the federal grants registry and the public website of Agtech company Syngenta. Searches were performed using a variety of relevant keywords, including (“fludioxonil OR phenylpyrrole”) AND (“oxidative stress” OR “environmental damage” OR “PFAS” OR “Carcinogenic” OR “Apoptosis” OR “glutathione/GSH” OR “Genotoxicity” OR “Autoimmune/autoimmunity” OR “Endocrine disruption/disruptor”). Synonyms and abbreviations for key words were likewise included in search strategies.

Criteria for inclusion involved reliability of the source literature and unambiguous, quantifiable evidence for damage to the environment or to human health. Reports involving structural studies were additionally validated *via* duplication of the work with the full cooperation of those report’s authors.

## Fludioxonil Persistence in Soil, Surface and Groundwater

### Environmental fate of fludioxonil

When fludioxonil was originally engineered from pyrrolnitrin, the intention was solely to improve resistance to environmental degradation. In this regard, its inventors were conditionally successful as fludioxonil lasted for days on surfaces to which it was applied. Fludioxonil that infiltrates surrounding soil, and is thus shielded from sunlight, may linger for much longer, however (Bermudez-Couso et al., 2007; Lewis et al., 2016) (See ‘Parameters and mechanisms of fludioxonil persistence’). Of note, while the toxicity of many pesticides is reduced by environmental forces such as the UV rays in sunlight, fludioxonil seems to behave in the opposite manner (Lassalle et al., 2015). Lassalle et al. (2015) reported that not only is fludioxonil largely transformed into two primary photolysis breakdown products by UV irradiation, the products generated in this manner are approximately 100-fold more toxic than the parent compound in a standard *Vibrio fischeri* toxicity assay.

**Figure 3:**
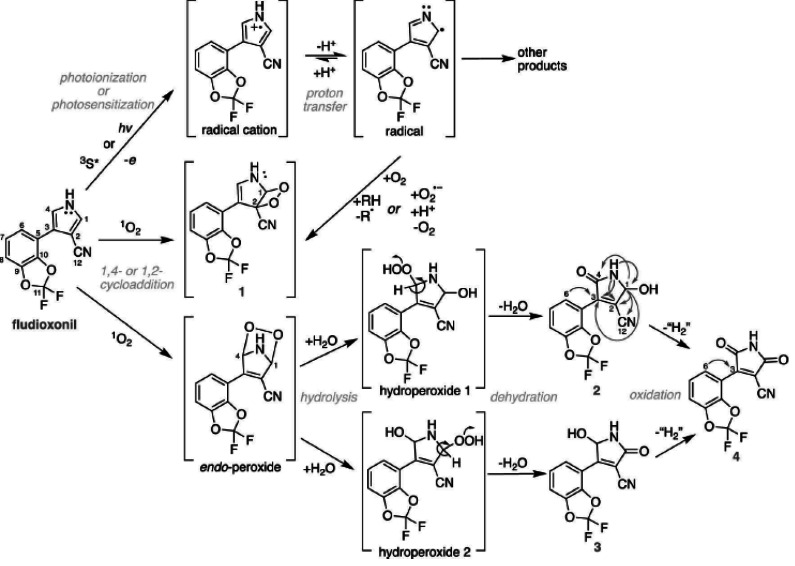
Theoretical model of UV induced oxidation/hydrolysis of fludioxonil replicated with permission from the McNeill lab (Apell, Pflug & McNeill, 2019).

While Lassalle et al. (2015) assessed fludioxonil’s susceptibility to photolysis accurately, predictions regarding the structure of the breakdown products were less firm. Apell, Pflug & McNeill (2019), using a combination of mass spectrometry and nuclear magnetic resonance, more accurately assessed the breakdown pathway of this fungicide (Fig. 3). They found that each molecule of fludioxonil may be photo-activated by UV wavelengths in sunlight, inducing its pyrrole ring to bind O_2_. Further reaction in the presence of water may create an intermediate with one hydroxyl group and one carbonyl group in the 2 and 5 positions. A portion of this primary breakdown product (7–12%) will further dehydrogenate spontaneously to create a dioxo-pyrrole (structure #4 in Fig. 3), a substance which has been confirmed to exist in soil samples taken from agricultural land where fludioxonil was applied (EFSA, 2007). These more toxic breakdown products may be of far greater concern than fludioxonil itself.

In the US, regulations are in place through the Office of Chemical Safety and Pollution Prevention to prohibit use of fludioxonil adjacent to bodies of water and forbid the discharge of fludioxonil-containing waste into lakes, streams, ponds, estuaries and oceans (Giles-Parker, 2025). However, due to the recent surge in nationwide flooding, the proper discharge of fludioxonil laden runoff in agricultural settings cannot reasonably or reliably be ensured. During periods of increased flooding and soil erosion, ecologists are detecting levels of fludioxonil in runoff streams sufficiently high to violate established guidelines, sometimes by a factor of up to a thousand (Aamlid et al., 2021).

**Figure 4:**
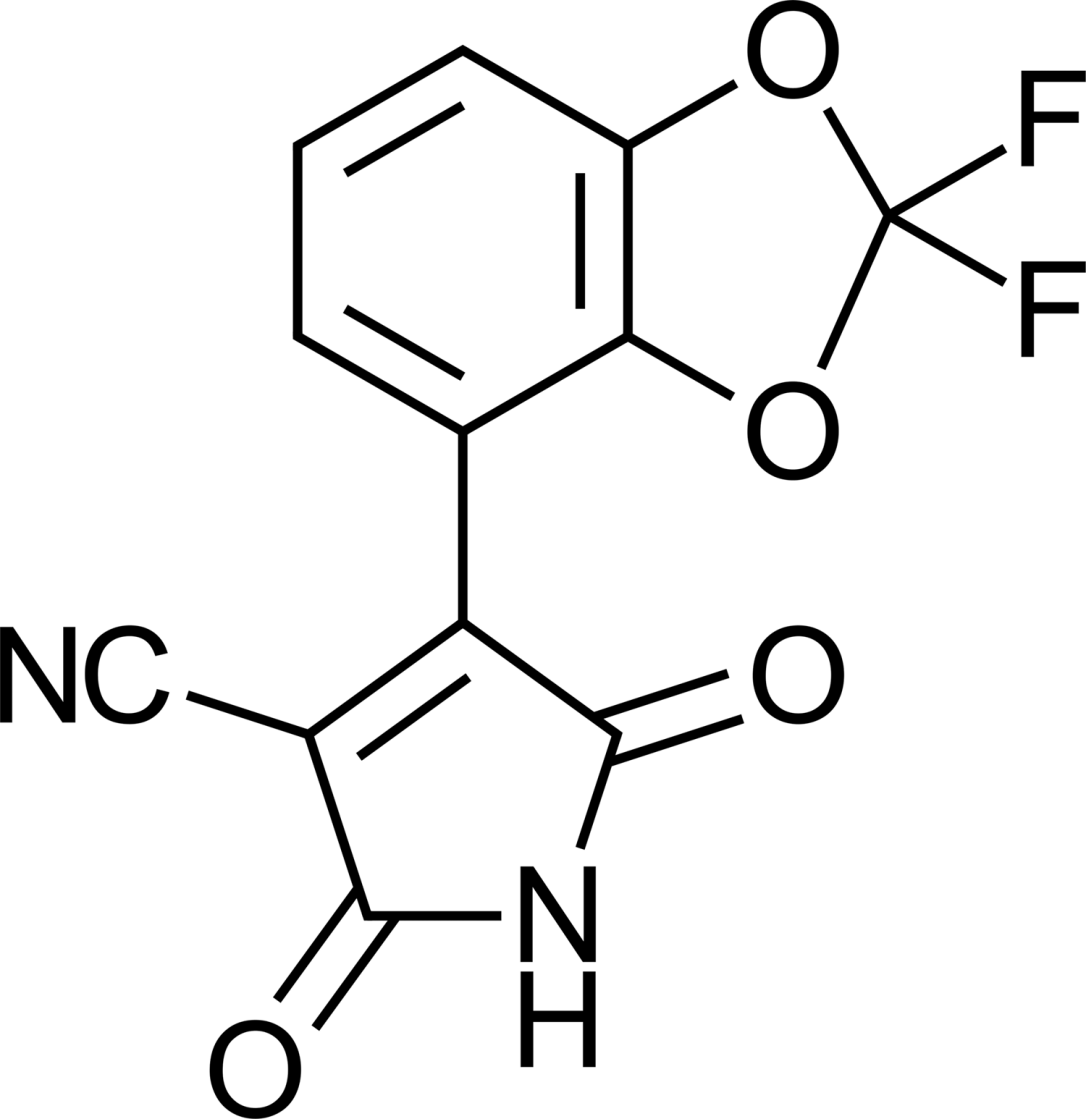
Maleimide that fludioxonil turns into CGA 265378.

While fludioxonil itself is widely considered to have low mobility in most soil types, there remains significant potential for particle-bound transport to occur due to erosion and soil run off. Green (2021) and Tian et al. (2021) reported markedly higher than predicted levels of fludioxonil in agricultural watersheds, at times reaching up to 10 ppm. The capacity of the primary breakdown products of fludioxonil (structures #2 and #3 in Fig. 3) to mobilize from the soil into water tables has not been determined, but the European Food Safety Authority (EFSA) noted that the secondary breakdown product, the dioxo pyrrole CGA 265378 (Fig. 3, structure #4 and Fig. 4), becomes highly mobile in soil (EFSA, 2007), thus reaching, and potentially damaging aquatic environments and groundwater reservoirs. This is especially troubling considering that from 2010 to 2015 groundwater use in the US increased by 8.3% while surface water use declined by 13.9%. It is notable that no less than 38% of the US population currently depends upon groundwater reservoirs for its drinking water supply (Dieter et al., 2018).

### Parameters and mechanisms of fludioxonil persistence

Fludioxonil is considered to be highly persistent once this fungicide penetrates beneath the top layer of soil where it is protected from UV rays (Bermudez-Couso et al., 2007), possessing, in some cases, a half-life of one or moreyears (Table 1). It similarly seems to have a capacity to linger in surface waters and sediments over time. There has long been concern regarding fludioxonil’s potential to leach into groundwater (Navarro et al., 2011; Smalling et al., 2018), especially as this soil-sequestered reservoir of toxic potential accumulates. As an example, Garretson (2019) found that fludioxonil was present in a domestic well in Fresno County as part of the Groundwater Protection Program’s groundwater monitoring study (1999–present). In this well, fludioxonil was detected at increasing concentrations for the past three consecutive years, providing evidence that this fungicide might both accumulate in agricultural soils and leach into groundwater at an accelerating pace.

**Table 1:** Half-life of fludioxonil under different environmental conditions.

Environment	Half-life	Citation
Crop surfaces	35–45 days	Yao et al. (2021)
Soil surface	6–12 days	Zhang et al. (2018)
Shallow soil	87–228 days	USEPA/OPPTS (2012)
Deeper soil	140–350 days	Davies et al. (2013)
Water	450–700 days	MacBean (2008–2010)
Sediments	120 days	Choi et al. (2025)

Instances of groundwater contamination are likely to increase as fludioxonil accumulates in soil over time, a situation potentially exacerbated by the fluorine introduced into its structure by Ciba Geigy (Fig. 2) which rendered fludioxonil a perfluoroalkyl substance (PFAS) (Rasmusson & Fagerlund, 2024) or, as it is frequently referred to, a forever chemical. The effect of fludioxonil’s fluorination upon persistence in the environment has not been adequately studied, and the stability of its primary, more toxic breakdown products is also unknown, but a wide range of PFAS are being banned throughout the world for related concerns (EHN, 2024). The cumulative deleterious effect of PFAS on human health is only recently being appreciated (eBioMedicine, 2023; Fenton et al., 2021).

Fludioxonil persists not only in the environment, but also upon the surfaces of agricultural products, which it is often applied to post-harvest. In between picking and packaging items of produce may undergo a coating step involving concentrated fludioxonil. Upon drying, this residue is very difficult, if not impossible, to remove- washing and brushing steps have been found to have little effect and the efficacy of the preservative can linger on food surfaces for up to seven months (Xiao & Boal, 2009).

### Ecological effects

For a fungicide postulated to act with high specificity, fludioxonil is surprisingly toxic to numerous varieties of organisms both aquatic and terrestrial (Holzl, 2017). Against many of these organisms fludioxonil is categorized as a class 1 toxin, (the most toxic designation), and this has led to calls from limnologists/ecologists and Consumer Reports for a complete ban on its use near streams, rivers and lakes. The US EPA itself recognized in its 2017 risk assessment the seriousness of the threat fludioxonil poses to listed fish, aquatic invertebrates, birds, mammals, and aquatic and terrestrial plants, which warranted full biological evaluation and ESA Section 7 consultation. Phytoplankton are particularly at risk, with fludioxonil found to adversely impact growth, photosynthetic activity, oxidative stress, and cell morphology in *Chlorella vulgaris,* culminating in apoptosis (Liu et al., 2022) while similar outcomes were observed for chlorophyll-bearing diatoms (Syngenta, 2022). These observations indicate a potential for significant ecological disruption, considering the critical roles phytoplankton play as the foundations for oceanic food chains (Brander & Kiørboe, 2020), biogeochemical cycles and nutrient (re)cycling (Naselli-Flores & Padisak, 2023), carbon fixation and O_2_ production (Lei et al., 2025).

Off-target terrestrial organisms have been shown to be sensitive to fludioxonil-initiated toxicity, with data drawn from grapevines (Petit et al., 2008), *Caenorhabditis elegans (C. elegans)* (Velki et al., 2019; Choi et al., 2025), South American toads (Svartz, Acquaroni & Perez-Coll, 2018), cultured rat and mouse cells (Graiet et al., 2022; Lee et al., 2021), bovine mammary cells (Kim et al., 2025a) and human lymphocytes (Antonopoulou et al., 2025) and cardiomyocytes (Lee et al., 2021). The capacity of fludioxonil to trigger an immune response and reactive nitrogen species (RNS) stress in honey bee populations (Bartling et al., 2021) may correlate with premature death and colony collapse as well (Olgun, Dayıoğlu & Taskiran, 2020). Recent work has shown that fludioxonil toxicity affects a zebrafish (Danio rerio) model system through oxidative stress, mitochondrial dysfunction, impairment of the vascular system and neurogenesis in larvae (LD50 = 1 ppm) (Kim et al., 2025b). Wang et al. (2020) found that fludioxonil toxicity was synergistic with other pesticides in disrupting embryonic development in *D. rerio.* In field observations, amphibian populations were found to be significantly susceptible to pesticide mixtures, especially during developmental stages of growth (Svartz, Acquaroni & Perez-Coll, 2018; Svartz, Meijide & Perez Coll, 2016). This is particularly relevant, given that in modern agricultural practice pesticides tend to be applied in concert, not individually. Such concerns may also extend to species of granivorous birds as these have been observed to feed extensively upon fungicide-treated grain laid upon agricultural land and are vulnerable to the effect of pesticide mixtures (De Montaigu & Goulson, 2022).

Soil resident nematodes such as *C. elegans* are affected physiologically by exposure to fludioxonil, suffering deficits in growth, lifespan, and reproductive capacity (Choi et al., 2025). Overall organismal health is affected as individual cells show elevated ROS levels upon fludioxonil treatment and at the neuronal level *C. elegans* displayed dopaminergic neurodegeneration and aberrant motility and behavior, indicating a multi-faceted toxic effect upon this non-target organism (Choi et al., 2025). Given the variety of organisms described here which have proven to be adversely affected by fludioxonil, or more likely, the breakdown product(s) of fludioxonil, it may be prudent to further investigate the persistence of these breakdown products in the soil, particularly because these toxic products remain PFAS and may prove intractable to any further degradation.

### The biochemistry of an ecological hazard

The fact that CGA 265378 is a dioxo-pyrrole, commonly referred to as a maleimide, may be relevant to many of the observed biological effects associated with fludioxonil. Kim et al. (2007b) demonstrated that disruption of glutathione (GSH) homeostasis (which serves to buffer nitrosative, oxidative and aldehydic stressors) in fungi synergistically enhances the activity of fludioxonil. This data suggests that fludioxonil attacks cells by either disrupting GSH, driving up one or more of those chemical stressors, or both (Kim et al., 2007b). Disruption of electron transport in mitochondria, and concomitant release of reactive oxygen species (ROS) (Chen et al., 2024), is generally in keeping with the mechanism originally proposed for pyrrolnitrin (Tripathi & Gottlieb, 1969), the antibiotic fludioxonil was derived from, but at this point it is possible to assert a more specific mechanism. It has long been known that depletion of glutathione is causal for accumulation of chemical stressors (Vaziri et al., 2000) and maleimides (similar to the dioxo-pyrrole breakdown product CGA 265378) are highly reactive with biological thiols present in the reactive centers of many regulatory proteins, enzymes and GSH as well (Renoux et al., 2017; Block et al., 1988). In the intracellular milieu, maleimides bind to these reduced thiols irreversibly and with great avidity, disabling function (Babanyinah et al., 2024). Intriguingly, our own research confirmed that fungi treated with fludioxonil were rapidly depleted of all detectable GSH in a GSH-Glo^tm^ assay (unpublished data). A mechanism based upon depletion of GSH is also supported by the observation that N-acetyl cysteine (NAC) repletion (commonly used to enable/promote GSH regeneration) prevents many of the observed toxic effects of fludioxonil (Graiet et al., 2022). Glutathione depletion *via* fludioxonil may thus comprise a key mechanism for its toxicity in living organisms.

It should be emphasized here that the biological functions of GSH are critically important as this tripeptide serves as the master anti-oxidant in animals, plants and fungi, protecting them from oxidative damage derived from stress, pollution and disease (Labarrere & Kassab, 2022; Townsend, Tew & Tapiero, 2003). Glutathione acts by reacting with oxidizing elements that may otherwise deactivate critical regulatory proteins and damage DNA (Hammond, Lee & Ballatori, 2001; Chatterjee, 2013). The mechanism of action for every major class of pesticides involves some induction of ROS and/or RNS (Sule, Condon & Gomes, 2022), chemical stressors which would be subject to neutralization by glutathione. This may explain why fludioxonil has been recognized for its capacity to act synergistically with other pesticide preparations (Wang et al., 2020).

If fludioxonil toxicity is based, fully or partially, upon the capacity of maleimide breakdown product(s) to damage biological thiols and deplete GSH, that may explain why studies in lab environments, where UV photolysis is not a concern, tend to support postulates that fludioxonil is non-toxic, while field experiments and experiments in which UV light is present (to permit photosynthesis for example) tend to yield data indicative of a potent toxic effect (Liu et al., 2022).

### Residential exposure

Fungicides such as Medallion and Maxim XL, marketed currently for use in landscaping, golf courses, residential lawns, sports fields, parks, and playgrounds, contain 11–50% fludioxonil depending upon their specific formulation. This regularly places an exceptionally concentrated form of this pesticide proximal to vulnerable populations like children and the elderly, not to mention the workers who handle it (Prechsl et al., 2022). Further, fludioxonil has been registered and patented for incorporation into mold- and moisture-resistant drywall products (Cornish et al., 2006) and is included as a mold inhibitor in plastic wares for household use. Rural residences increasingly take their tap water from groundwater wells, posing a greater risk of exposure to pesticide residues to these communities as groundwater becomes contaminated (Dieter et al., 2018; Garretson, 2019). Fludioxonil’s presence in our daily lives is approaching ubiquitous, which is concerning considering its photolysis by the simple convergence of sunlight and moisture produces maleimide-containing breakdown products with the potential to debilitate cellular oxidative stress defenses (GSH) (Zhou et al., 2020).

## Fludioxonil and Human Health

### Oxidative damage

It has long been established that most, if not all, pesticides initiate oxidative damage either directly or as a side effect of their mechanism of toxicity (Sule, Condon & Gomes, 2022; Abdollahi et al., 2004). These effects are found not just within the cells of target fungal organisms, but also in human lymphocytes (Lee, Hwang & Choi, 2019), cultured neuronal and glial cells (Coleman et al., 2012), and cultured cardiomyocytes (Seong et al., 2024) following pesticide exposure. Some pesticides, including fludioxonil, were found to exert an inhibitory effect upon the cellular enzymes that protect against oxidative stress, such as superoxide dismutase (SOD) (Karadag & Ozhan, 2015; Wang et al., 2020). Superoxide is produced as a by-product of oxygen metabolism and, if not regulated by protective enzymes like SOD, may mediate significant damage to a variety of cellular targets including DNA, proteins and lipids (Bucher, 2019). While many pesticides inhibit SOD competitively, fludioxonil inhibits this enzyme non-competitively (Karadag & Bilgin, 2010), an important point since SOD activity requires the activity of a susceptible surface thiol, cys111. Irreversible binding of the thiol of cys111 results in non-competitive inhibition of activity (Okado-Matsumoto, Guan & Fridovich, 2006). A Micheal reaction between the thiol of cys111 and breakdown product CGA265378 stands as a likely model of fludioxonil’s inhibition of SOD, given the capacity of this pesticide to degrade into a thiol-reactive maleimide (Apell, Pflug & McNeill, 2019).

It is of interest that Brandhorst et al. (2019) determined that thiols were primary targets involved in the mechanism by which fludioxonil functions. While fludioxonil treatment results in oxidative stress within fungi, it appears that oxidative stress is not primarily responsible for the observed toxicity. The fungal regulatory kinase targeted by fludioxonil is affected through a crucial reactive thiol and it was determined that while a broad panel of oxidative stressors were insufficient to replicate the effects of fludioxonil toxicity, an aldehydic stressor that binds thiols irreversibly was able to mimic the toxic effect of fludioxonil in fungi (Brandhorst et al., 2019). It may be relevant that depletion of GSH permits aldehydic stressors to accumulate naturally as a by-product of glycolysis (*e.g.*, methylglyoxal) (Schalkwijk & Stehouwer, 2020).

Oxidative stress is often the result of damage to the mitochondria, being derived from an unregulated loss of mitochondrial membrane potential (MMP) and a failure to couple MMP to adenosine triphosphate (ATP) production (Xu, Pang & Fan, 2025). Human glial cells and neuronal cells incubated with fludioxonil demonstrated decreased MMP and ATP production at concentrations well below those otherwise considered toxic (Coleman et al., 2012). It is likely that current regulations failed to take into account this type of health damage. Rat glioma cells similarly exposed showed oxidative stress leading to cell cycle arrest, cytoskeletal degradation, DNA damage and apoptosis (Graiet et al., 2022). These effects occur in conjunction with a depletion of cellular thiol levels which make up the backbone of any cellular resistance to oxidative stress (Ulrich & Jakob, 2019).

**Figure 5:**
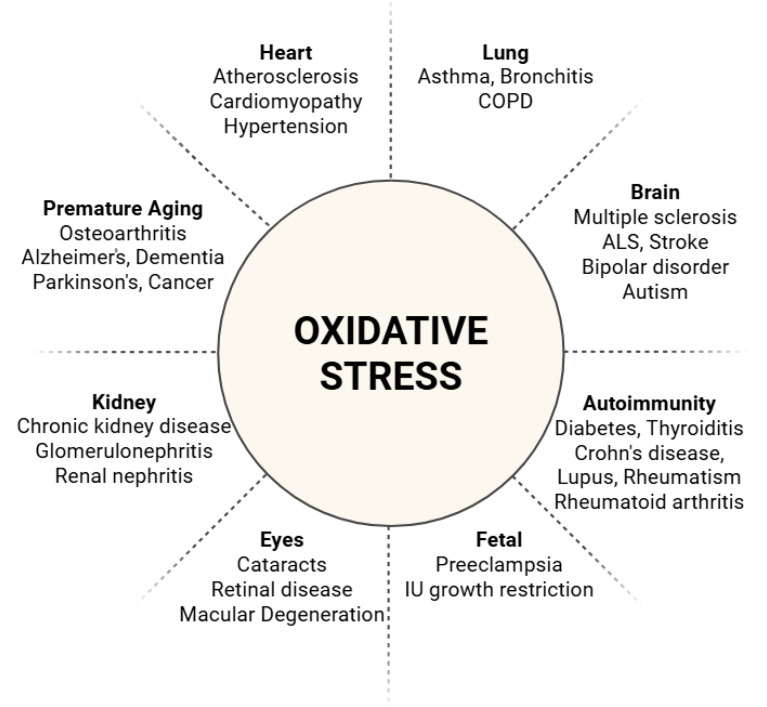
Disease states resultant from oxidative stress. Oxidative stress affects human health, inducing inflammation, premature aging, and biasing the immune system towards autoimmunity. This leads to an extensive list of disease states affecting multiple organs. (Kıran, Otlu & Karabulut, 2023; Pham-Huy, He & Huy, 2008) Created in: Biorender.

The confirmatory discovery that fludioxonil exposure depletes GSH (thiols) in mammalian glial cells is worth addressing. This replication of the GSH depletion effect in mammalian cells may validate the theory that maleimide-based, irreversible binding of thiols takes place in off-target organisms. Glutathione depletion is recognized as a damaging form of toxicity by modern toxicology (Dethlefsen et al., 1986; Kanz et al., 2003). The potential for the joint effects of oxidative damage and GSH depletion to synergistically impact human health needs to be taken into account during regulatory consideration. Further, chronic dysregulation of the balance between oxidants and anti-oxidants has been associated with a long list of autoimmune disease states (including systemic lupus erythematosus, rheumatoid arthritis (RA), and multiple sclerosis (MS)) (Perricone, De Carolis & Perricone, 2009), inflammatory disorders (including metabolic syndrome and hepatic disease) (Hristov, 2022), degenerative diseases of lung and heart function (including coronary artery disease and COPD) (Matuz-Mares et al., 2021), neurological diseases (including Parkinson’s and Alzheimer’s) (Hristov, 2022) and increased cancer risk (Pizzino et al., 2017) (Fig. 5).

### Immunotoxicity

Fludioxonil was shown to exert adverse effects upon populations of T and B lymphocytes (Lee, Hwang & Choi, 2019), preventing their maturation and triggering apoptosis, potentially due to the role GSH normally plays in the maturation and differentiation of these cell types (Liang et al., 1991). Fludioxonil decreased the viability of these immune cells co-incident with a fall in MMP. Pro-apoptotic proteins accumulating in these immune cells as a result of fludioxonil exposure led to arrest of their cell cycle, eventually resulting in apoptosis (Lee, Hwang & Choi, 2019). This result could occur at concentrations of fludioxonil achievable *via* consumption of produce with lower than the MRL of fludioxonil, as determined by Medjakovic et al. (2014). Damage done to these cell types by fludioxonil (or more likely its breakdown products) may enhance a toxic insult by rendering the body susceptible to subsequent threats through depletion of GSH, which induces immune dysfunction (Droge & Breitkreutz, 2000) and prevents both dendritic cell maturation (Kim et al., 2007a) and priming of T cells (Mak et al., 2017). In addition, an imbalance in the ratio between oxidative potential and antioxidant capacity may theoretically predispose the body to autoimmune malfunction (Zifman et al., 2008) and lead to a variety of autoimmune pathologies. This is noteworthy considering recent increases in the incidence of autoimmune diseases such as diabetes, celiac disease, inflammatory bowel disease and multiple sclerosis) (Miller, 2023).

### Endocrine disruption

In a broad study of pesticide toxicity, fludioxonil was one of 8 pesticides determined to be capable of antagonizing androgen receptors (antiandrogenic activity) in a human breast cancer cell model system (Orton et al., 2011; Orton et al., 2012). This effect was found to be not only additive with other similar pesticides, but synergistic, working in tandem to further amplify reproductive dysfunction. This synergy may be derived from GSH depletion, as this antioxidant serves to detoxify many endocrine-disrupting chemicals (*e.g.*, benzophenone, p-octylphenol, and tributyltin chloride) in both human and mouse model systems (Kato et al., 2006).

Teng et al. (2013) reported that fludioxonil had the capacity to act as an endocrine disrupting pesticide (EDP) as evidenced in androgen and estrogen receptors on the surface of human breast cancer cell lines and prostate cancer cell lines. Kugathas et al. (2016) noted that fludioxonil suppressed prostaglandin D2, attributing this effect to binding in the COX-2 active site, raising concerns regarding malformation of fetal sexual development. Orton et al. (2011) blames the widespread decline in male reproductive health on the EDPs in our diet, reporting that fludioxonil had one of the strongest anti-androgenic effects *in vitro*. Scholze et al. (2020) found that the antiandrogenic effects of fludioxonil lead to disfigurement of the sexual organs in a rat model system, while Svartz, Meijide & Perez Coll (2016) found analogous disfigurement in an amphibian model at concentrations of fludioxonil achievable *via* consumption of produce with lower than the MRL of fludioxonil. A correlation between fluorination and EDP has been reported very recently, but authors were not able to establish a clear mechanism (Liu et al., 2025).

### Hepatotoxicity

Lasch et al. (2021) demonstrated the capacity of fludioxonil to induce non-steatotic hepatic damage so thoroughly that this fungicide has been employed as a positive control in examinations of the effect that other, less toxic pesticides have upon liver tissues. This effect upon the liver is in part derived from the capacity of fludioxonil to inhibit certain metabolic enzymes like CYP3A4 (Lasch et al., 2021). It is well-known that chronic toxification of the liver due to an ubiquitous fungicide might adversely affect human health systemically (Lu, 2020). It is also commonly understood that liver health is intrinsically dependent upon maintenance of GSH, which drives the liver’s primary function of toxin detoxification. This constitutes the basis for toxicologists considering GSH depleters to be particularly dangerous in that these compounds can potentiate the effects of many other toxins (Labarrere & Kassab, 2022).

Experiments in human liver microsomes demonstrated that fludioxonil that reaches the liver undergoes the same hydroxylation breakdown reactions that Apell, Pflug & McNeill (2019) described occurring upon UV exposure in the field and Lassalle et al. (2015) detected occurring on the skin of pesticide treated grapes (Huang, Law & Leung, 2023). The metabolites resulting from liver “detoxification” reactions were analyzed by mass spectrometry and found to be similar in structure, if not identical, to fludioxonil photolysis products #2 and #3 (Fig. 3). This means that fludioxonil doesn’t have to be broken down *via* UV prior to ingestion to constitute a threat to human health. *In silico* toxicological analyses predict that these breakdown products may be more toxic than parent fludioxonil (Huang, Law & Leung, 2023), though this prediction did not specifically take into account the capacity of maleimide-containing molecules to induce GSH depletion. Thus, fludioxonil that has not been degraded by UV exposure may still be converted into harmful metabolites during the process of concentrating/processing in the liver. That this conversion process takes place is sufficiently established that these metabolites are currently recommended as serving as biomarkers for pesticide exposure (Huang, Law & Leung, 2023).

### Neurotoxicity

Fludioxonil is an activator of the aryl hydrocarbon receptor (AHR) (Medjakovic et al., 2014) (EC_50_ value of 0.42 µM) which has been implicated in the induction of neurotoxicity effects. In *in vitro* studies, fludioxonil has been shown to induce neurotoxicity in human neuronal and glial cell lines. Medjakovic et al. (2014) determined that consumption of produce with lower than the MRL of fludioxonil might lead to serum concentrations well above the EC_50_ value for fludioxonil binding to AHR. Graiet et al. (2022) reported that fludioxonil above five ppm elicited cytoskeleton disruption, DNA damage, cell cycle arrest, loss of MMP and apoptosis, each to a significant degree, in a glioma model of neurotoxicity. Each of these indicators of toxicity was markedly reversed by co-treatment with NAC (the biological thiol that plays a key role in restoring depleted GSH levels) (Atkuri et al., 2007). It is also notable that GSH depletion is known to directly induce each of these toxic indicators: cytoskeletal disruption (Zepeta-Flores et al., 2018), DNA damage (Gokce et al., 2009), cell cycle arrest (Odom et al., 2009) and apoptosis (Franco & Cidlowski, 2009; Roy & Paira, 2024) and serves to exacerbate loss of MMP by allowing unchecked ROS to damage mitochondrial components (Thomas, Shay & Hagen, 2019; Roy & Paira, 2024).

### Carcinogenesis

Triple-negative human breast cancer cells treated *in vitro* with fludioxonil developed into polyploid giant cancer cells (PGCCs). These PGCCs showed (1) increased motility (metastasis), (2) became resistant to anticancer drugs and (3) daughter cells displayed stem-cell like qualities enhancing their capacity to self-renew, differentiate, and initiate tumor growth, contributing to tumor progression and recurrence (Go et al., 2024). Arnaud et al. (2021) found that while fludioxonil by itself did not induce carcinogenic effects, in conjunction with other pesticides this fungicide initiated carcinogenic effects in human colon cell lines *via* both genotoxic (damaging to DNA) and non-genotoxic mechanisms. This was observed at concentrations of fludioxonil achievable *via* consumption of produce with lower than the MRL of fludioxonil. This discovery is highly relevant, as pesticides are rarely tested for toxicity in mixtures and yet, due to the nature of modern agricultural practices, pesticides are rarely applied to crops singly.

### Cardiotoxicity

Cardiovascular disease (CVD) currently accounts for 30% of human mortality and the prevalence of CVD has doubled in the last 30 years (Lindstrom et al., 2022). Cardiomyocyte damage is the primary pathophysiologic cause of cardiac dysfunctions including heart failure and myocardial infarction, thus environmental factors which directly impair the functioning of these contractile cells are of interest to human health (Chiong et al., 2011; Yu et al., 2023). In two different cardiomyocyte models, one rat, one human, fludioxonil was found to (1) enhance the expression of pro-apoptotic markers, (2) reduce oxygen consumption rate (OCR) and (3) trigger decreased MMP (Seong et al., 2024). Seong et al. (2024) suggested that a decrease in cardiomyocyte cell viability due to fludioxonil may theoretically be derived from elevated apoptotic changes and enhanced production of mitochondrial ROS proceeding from mitochondrial dysregulation. Thus, heavily correlated with oxidative stress and/or the depletion of cellular antioxidants.

## Conclusions

Based upon the reviewed evidence, risk managers and regulators may wish to consider that:

1. Sunlight and/or enzymes in the liver convert fludioxonil into breakdown products that have different characteristics, different reactivities and appear to be far more toxic than parent fludioxonil (Lassalle et al., 2015).
2. One of these breakdown products, CGA-265378, includes a dioxo pyrrole, also called a maleimide (Apell, Pflug & McNeill, 2019)—a toxic moiety that neutralizes biological thiols by binding to them permanently. Chlorothalonil, a fungicide that shares this mechanism of action is banned from being applied to food intended for human consumption in the European Union and is tightly regulated in the US (Bessi et al., 1999).
3. The toxic effect of fludioxonil is highly synergistic with other pesticides (Wei et al., 2021), a quality which needs to be considered with regard to its potentially toxic effects on non-target organisms (Wang et al., 2020).
4. The fact that breakdown product CGA-265378, unlike fludioxonil, becomes highly mobile in soil (EFSA, 2007) poses a significantly elevated threat to aquifers, especially considering increasing instances of hurricane and flooding and the capacity of PFAS to persist in the environment.

Appeals to the US EPA have been suspended pending two concerns: one, that the evidence that fludioxonil photolyzes into toxic breakdown products might not be reliable or robust (Lassalle et al., 2015; Apell, Pflug & McNeill, 2019) and: two, the US EPA cited a paucity of evidence linking GSH depletion to liver toxicity. To address the first of these concerns this review includes our lab’s verification/validation of prior structural studies employing the Mass Spectrometer facility of the UW-Madison Biotechnology Center and the NMR facility (NMRFAM) at the UW-Madison (Supplementary material). The initial observations of Lassalle et al. (2015) and the structural conclusions of Apell (Apell, Pflug & McNeill, 2019) were duplicated and duly verified by the expert staff collaborating at these facilities.

In addressing the second concern, toxicologists have considered GSH depletion a valid form of toxicity for decades (Dethlefsen et al., 1986; Kanz et al., 2003). Low GSH permits ROS to build up in tissues initiating oxidative damage (Lu, 2020) and GSH depletion potentiates essentially all other oxidative stressors. The list of disease states that are derived from oxidative damage is extensive, comprising inflammatory effects, premature aging of affected organs and the biasing of the immune system to drive auto-immune reactions (Fig. 5) (Tugba, Otlu & Karabulut, 2023; Pham-Huy, He & Huy, 2008).

As fungicides that act *via* GSH depletion are expressly forbidden for use as additives/coating for produce in the EU and tightly regulated in the US (*e.g.*, Chlorothalonil) (Bessi et al., 1999) it may be prudent to reevaluate the restrictions/allowances currently in place for this product. To conclude, given the risks fludioxonil poses to the environment in general and pollinators in particular, in addition to various toxic and endocrine disrupting effects, continued fludioxonil registration for direct use upon produce may pose adverse effects that must be weighed against its advantages as an agricultural tool.

##  Supplemental Information

10.7717/peerj.21290/supp-1 Supplemental Information 1 Materials and methods for MS dataset

10.7717/peerj.21290/supp-2 Supplemental Information 2 Validation of structure *via* MS

10.7717/peerj.21290/supp-3 Supplemental Information 3 Materials and methods for NMR

10.7717/peerj.21290/supp-4 Supplemental Information 4 Validation of structure by NMR

## Additional Information and Declarations

### Data Availability

The following information was supplied regarding data availability: This is a Literature Review.
