# Supplementary material for: Breakdown products of the fungicide Fludioxonil may account for observed environmental impact: potential implications for human health
Source: PeerJ. 2026 Jun 3;14:e21290. doi: 10.7717/peerj.21290 (PMC13242197; doi:10.7717/peerj.21290)
Supplement: Supplemental Information 1 [file peerj-14-21290-s001.docx]

*LC-HR-MS analysis*

The UV-treated fludioxonil prep at 1mg/ml in 40% acetonitrile [vol:vol] was profiled in its native form with 2µl injection for data dependent LC-HR-MS acquisitions under negative ionization using 1200 series LC system (Agilent) connected to hybrid linear ion trap-orbitrap mass spectrometer (LTQ-Orbitrap Elite™, Thermo Fisher Scientific) equipped with an HESI™ electrospray source. Chromatography prior to mass spectral acquisition was accomplished using InertSustain C18 column (3µM, 150x2.1mm, GL Sciences) kept at 35°C. HPLC system delivered solvents A: 0.1% Formic acid (pH 3) and B: 99.9% (v/v) acetonitrile with 0.1% (v/v) Formic acid (pH3) at 250 µL/min to load and elute compounds directly into the HESI-II probe with a primary gradient from 2% (v/v) B to 50% (v/v) B over 24 minutes, followed by rapid 50% (v/v) B to 95% (v/v) B in 3 minutes and concluded with 1 minute flash-out at 95% (v/v) B. The following source conditions were established for the most efficient ionization in the negative mode, source voltage: 2.5kV, gas temperature: 300°C, sheath gas flow: 35, aux gas flow: 7, S-Lens RF level: 40% and capillary temperature: 350°C. As compounds eluted from the HPLC-column/electrospray source survey MS scans were acquired in the Orbitrap with a resolution of 120,000 and mass range of 50 to 500 m/z. Manual chromatogram and spectral analysis was done using Qual Browser function in Thermo Xcalibur ver. 4.1.50 (Thermo Scientific).

*LC-MS/MS analysis*

The UV-treated fludioxonil prep at 1mg/ml in 40% acetonitrile [vol:vol] was used for MS/MS structural analysis of the most abundant and isomeric hydrolysates present with 20µl injection for data dependent LC-HR-MS-CID-MS/MS acquisitions under negative ionization using 1200 series LC system (Agilent) connected to hybrid linear ion trap-orbitrap mass spectrometer (LTQ-Orbitrap Elite™, Thermo Fisher Scientific) equipped with an HESI™ electrospray source. Chromatography prior to mass spectral acquisition was accomplished using InertSustain C18 column (3µM, 150x2.1mm, GL Sciences) kept at 35°C. HPLC system delivered solvents A: 0.1% Formic acid (pH 3) and B: 99.9% (v/v) acetonitrile with 0.1% (v/v) Formic acid (pH3) at 250 µL/min to load and elute compounds directly into the HESI-II probe with a primary gradient from 2% (v/v) B to 50% (v/v) B over 24 minutes, followed by rapid 50% (v/v) B to 95% (v/v) B in 3 minutes and concluded with 1 minute flash-out at 95% (v/v) B. The following source conditions were established for the most efficient ionization in the negative mode, source voltage: 2.5kV, gas temperature: 300°C, sheath gas flow: 35, aux gas flow: 7, S-Lens RF level: 40% and capillary temperature: 350°C. As compounds eluted from the HPLC-column/electrospray source survey MS scans were acquired in the Orbitrap with a resolution of 120,000 followed by a targeted CID-type MS/MS fragmentation into the Ion Trap (centroid data and normal scan rate) for 247 AMU, 279 AMU, 267 AMU and 277AMU ions detected in the MS1 scan from 50 to 500 m/z. Normalized collision energy of 40, isolation width of 2 m/z and activation time of 10 ms was applied. Manual chromatogram and spectral analysis was done using Qual Browser function in Thermo Xcalibur ver. 4.1.50 (Thermo Scientific).
