# Supplementary material for: Breakdown products of the fungicide Fludioxonil may account for observed environmental impact: potential implications for human health
Source: PeerJ. 2026 Jun 3;14:e21290. doi: 10.7717/peerj.21290 (PMC13242197; doi:10.7717/peerj.21290)
Supplement: Supplemental Information 2 [file peerj-14-21290-s002.pdf]

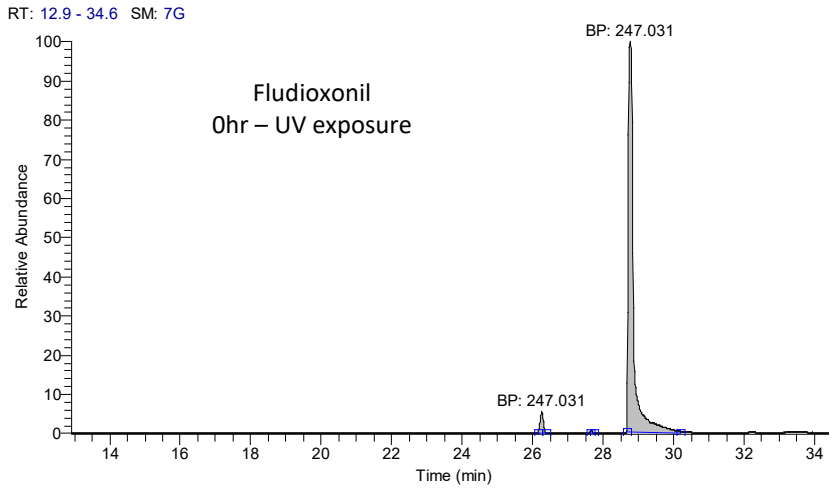

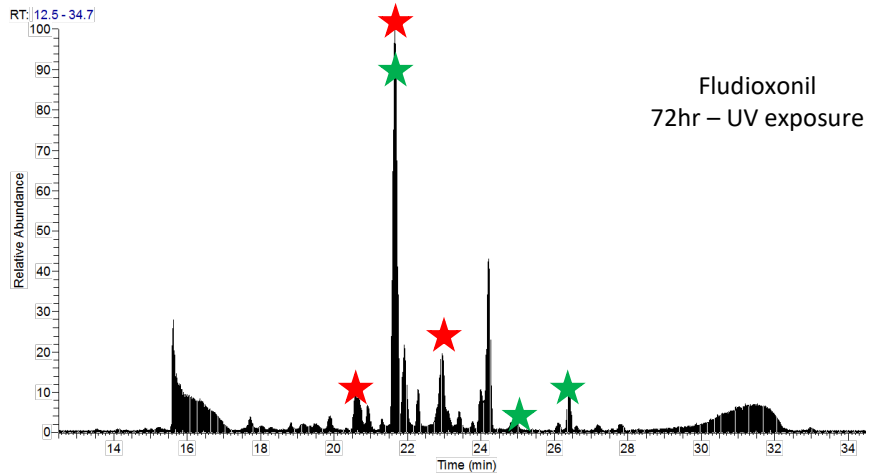

Extracted Ion Chromatogram [di-oxygenated Fludioxonil]

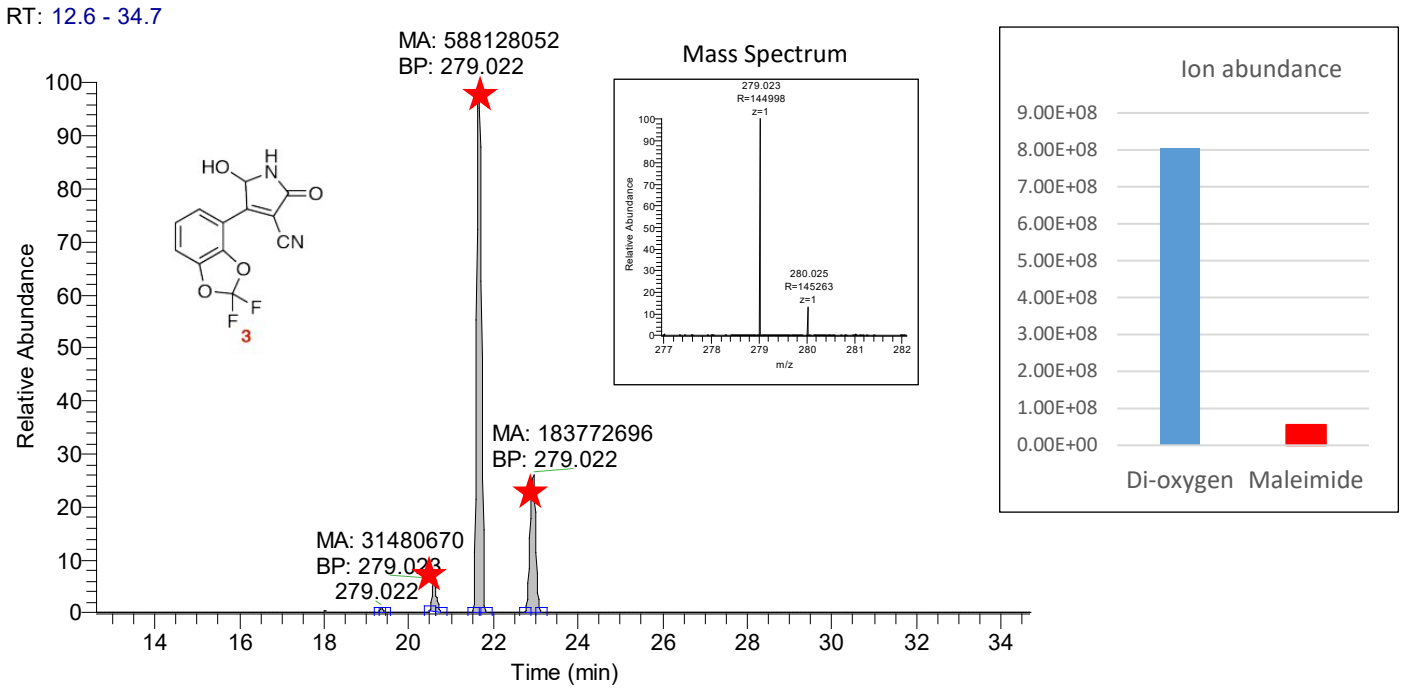

Extracted Ion Chromatogram [maleimide Fludioxonil]

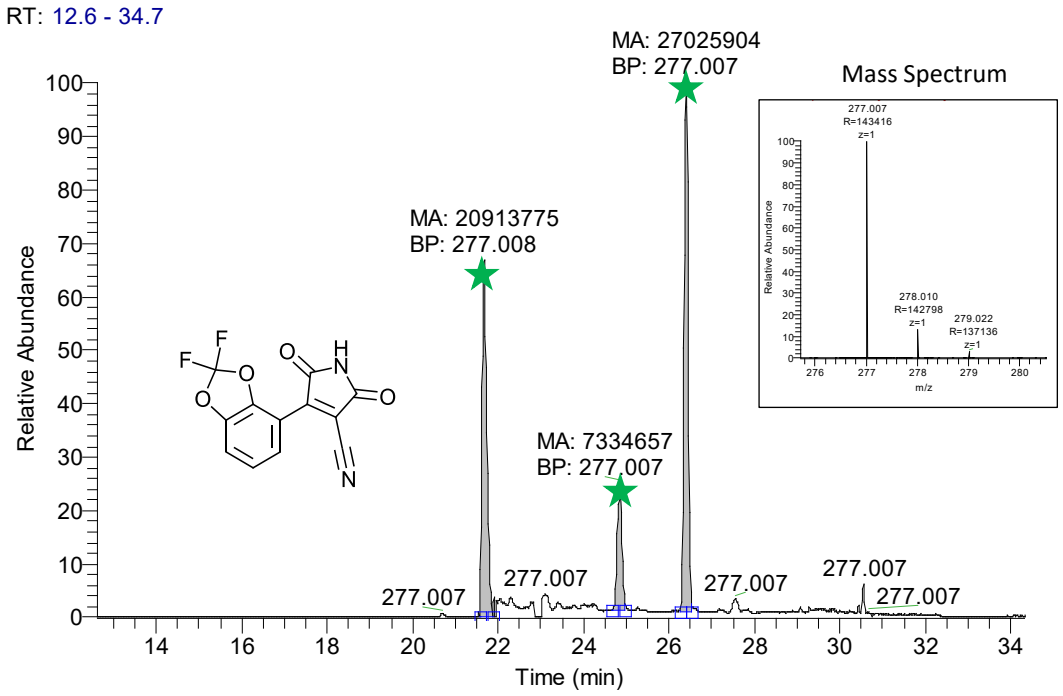

Orbitrap-Elite CID-type MS/MS

fludioxonil-72hr_cid-msms-targeted_12...
20ul inj of 1mg/ml

12/12/23 11:48:12

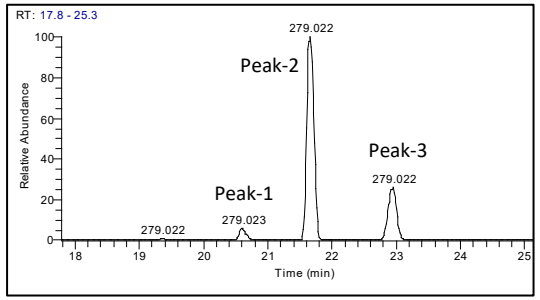

Peak-1

NL: 3.12E6
fludioxonil-72hr_cid-msms-targeted_121223_2#7953-8039 RT: 20.542-20.706 AV: 14 F: ITMS - c ESI r Full ms2 279.02@cid40.00 [75.00-300.00]

213.015
251.980
208.034
185.032
114.188
121.127
131.076
142.155
162.179
170.126
196.206
215.631
236.052
248.136
259.033

Peak-2

NL: 6.17E7
fludioxonil-72hr_cid-msms-targeted_121223_2#8412-8494 RT: 21.581-21.740 AV: 14 F: ITMS - c ESI r Full ms2 279.02@cid40.00 [75.00-300.00]

213.009
251.952
185.011
208.059
236.024
101.373
114.231
121.109
134.015
142.138
157.164
170.092
195.184
215.124
231.050
238.885
259.188

Peak-3

NL: 1.66E7
fludioxonil-72hr_cid-msms-targeted_121223_2#9004-9084 RT: 22.858-23.008 AV: 13 F: ITMS - c ESI r Full ms2 279.02@cid40.00 [75.00-300.00]

208.006
251.966
236.034
185.018
213.076
170.050
121.092
91.065
97.224
142.118
157.031
192.888
216.148
233.022
261.114

Relative Abundance

m/z

Orbitrap-Elite CID-type MS/MS

Fludioxonil-72hr_CID-MSMS-targeted_12...
20ul inj of 1mg/ml

12/12/23 11:48:12

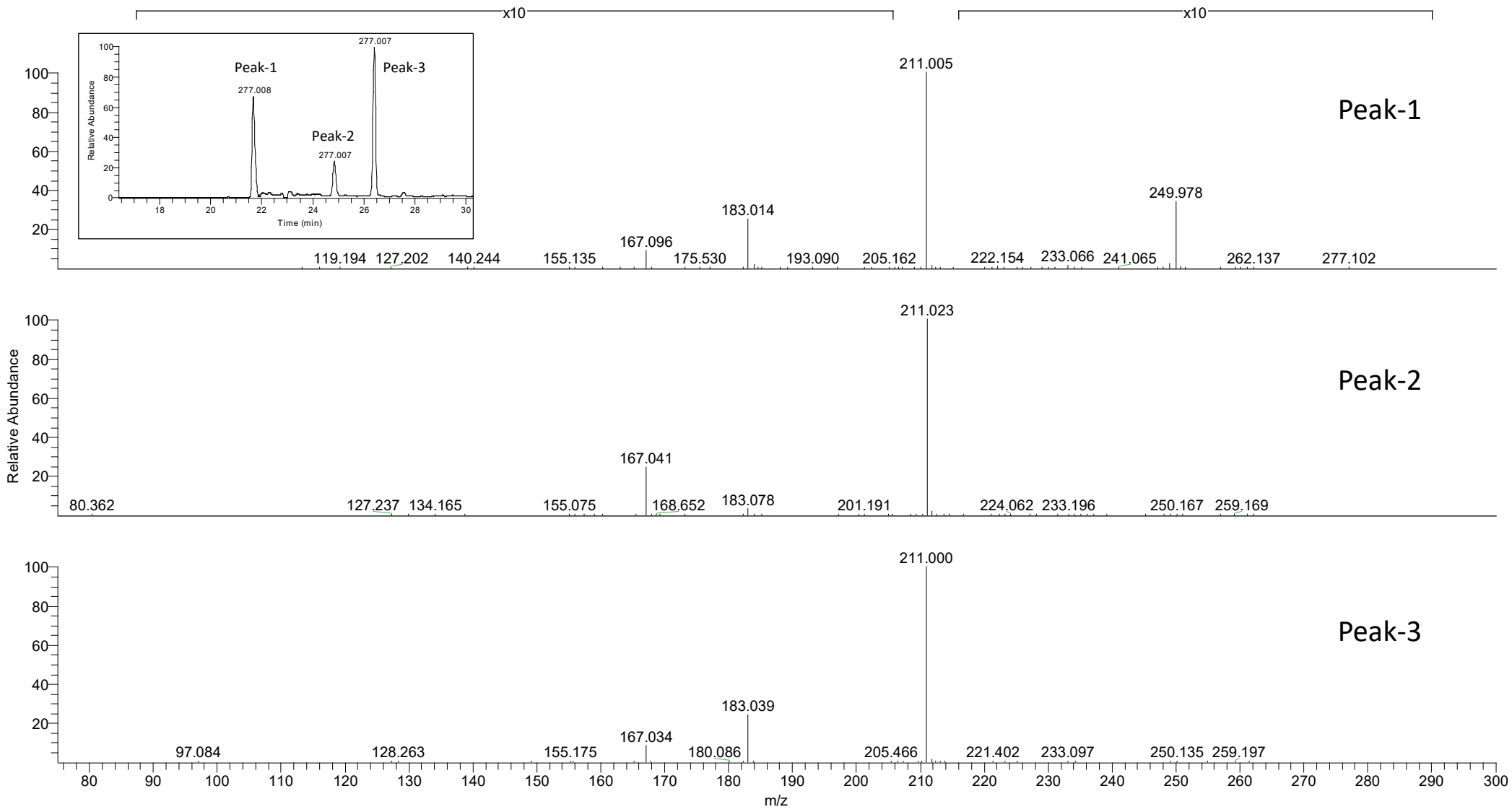

NL: 8.22E5
Fludioxonil-72hr_CID-MSMS-targeted_121223_2#8529-8615 RT: 21.817-21.982 AV: 15 F: ITMS - c ESI r Full ms2 277.02@cid40.00 [75.00-300.00]

NL: 2.30E6
Fludioxonil-72hr_CID-MSMS-targeted_121223_2#9823-9923 RT: 24.710-24.933 AV: 17 F: ITMS - c ESI r Full ms2 277.02@cid40.00 [75.00-300.00]

NL: 8.32E6
Fludioxonil-72hr_CID-MSMS-targeted_121223_2#10461-10547 RT: 26.287-26.472 AV: 15 F: ITMS - c ESI r Full ms2 277.02@cid40.00 [75.00-300.00]
