# Supplementary material for: Breakdown products of the fungicide Fludioxonil may account for observed environmental impact: potential implications for human health
Source: PeerJ. 2026 Jun 3;14:e21290. doi: 10.7717/peerj.21290 (PMC13242197; doi:10.7717/peerj.21290)
Supplement: Supplemental Information 3 [file peerj-14-21290-s003.docx]

The main products of the reaction were purified from the crude mixture by column chromatography on silica gel (10 to 50% EtOAc/hexane). The eluate was collected in 2-3ml fractions. The presence of purified products in the fractions was monitored by TLC. Tubes containing the same product were combined and the eluting solvent mixture evaporated under reduced pressure. The products were then redissolved in deuterated chloroform to prepare the samples for NMR data collection.

All experiments were recorded on Bruker Avance III spectrometers operating at 500, 600 and 750MHz (^1^H) and equipped with cryogenic probes. The temperature of the samples was regulated at 25°C throughout data collection.

In order to identify the purified products, one-dimensional (1D) ^1^H and ^13^C spectra (regular, DEPT-90 and DEPT-135), as well as two-dimensional (2D) ^1^H,^1^H-NOESY, ^1^H,^1^H-COSY, ^1^H,^1^H-TOCSY, ^1^H,^13^C-HSQC, ^1^H,^13^C-HSQC-TOCSY and ^1^H,^13^C-HMBC were recorded using standard Bruker parameters. In addition, 1D ^19^F spectra, as well as ^19^F-decoupled ^13^C spectra were recorded using a QCI-F Bruker cryoprobe.

The spectra were analyzed using Bruker Topspin (v3.5.7). 2D spectra were also processed with NMRPipe [F. Delaglio *et al.*] and analyzed using NMRFAM-SPARKY [Lee, W., Tonelli, M. & Markley, J. L. (2014)].

The compounds were identified by looking at the chemical shift of the peaks, the peak splitting patterns resulting from scalar coupling between nearby nuclei and the connectivity between peaks from the 2D spectra.

F. Delaglio *et al.*,(1965) NMRPipe: a multidimensional spectral processing system based on UNIX pipes. *J. Biomol. NMR* **6**, 277-293

Lee, W., Tonelli, M. & Markley, J. L. (2014) NMRFAM-SPARKY: enhanced software for biomolecular NMR spectroscopy. *Bioinformatics* **31**, 1325-1327, doi:10.1093/bioinformatics/btu830
