# Supplementary material for: Breakdown products of the fungicide Fludioxonil may account for observed environmental impact: potential implications for human health
Source: PeerJ. 2026 Jun 3;14:e21290. doi: 10.7717/peerj.21290 (PMC13242197; doi:10.7717/peerj.21290)

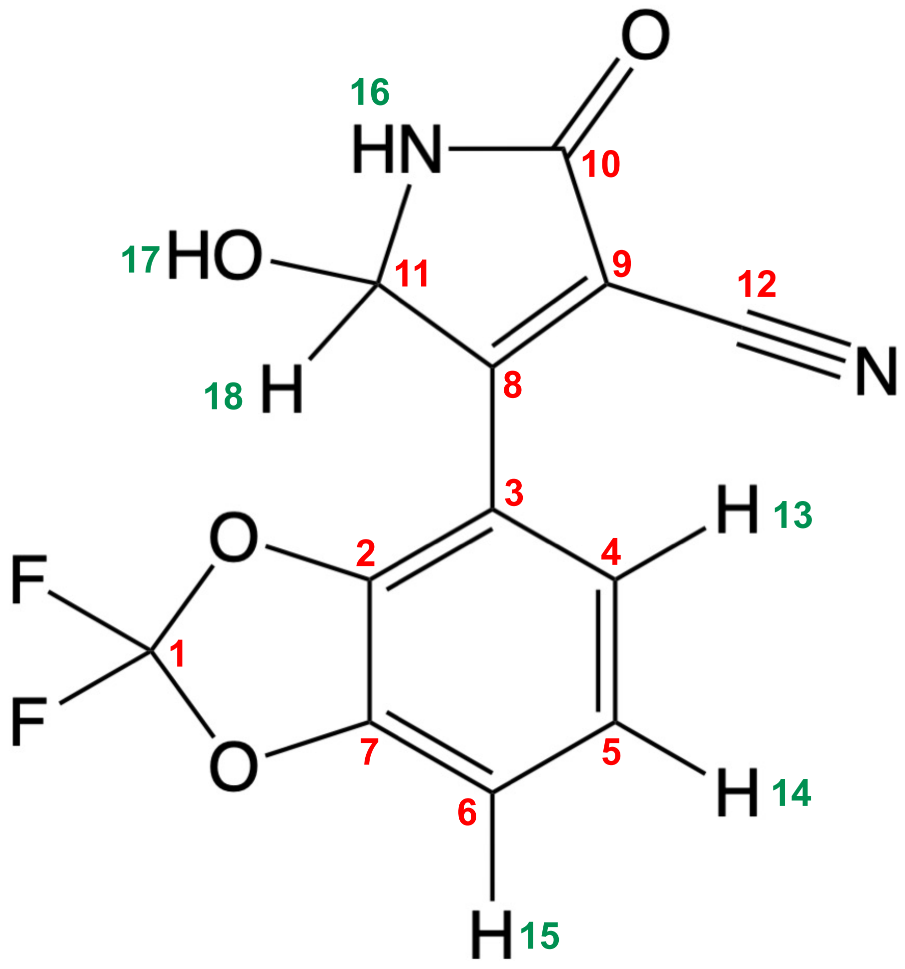

O
HN
16
10
17
HO
11
9
12
N
18
H
8
3
H
13
O
2
4
F
1
F
O
7
5
H
14
6
H
15

MW 280

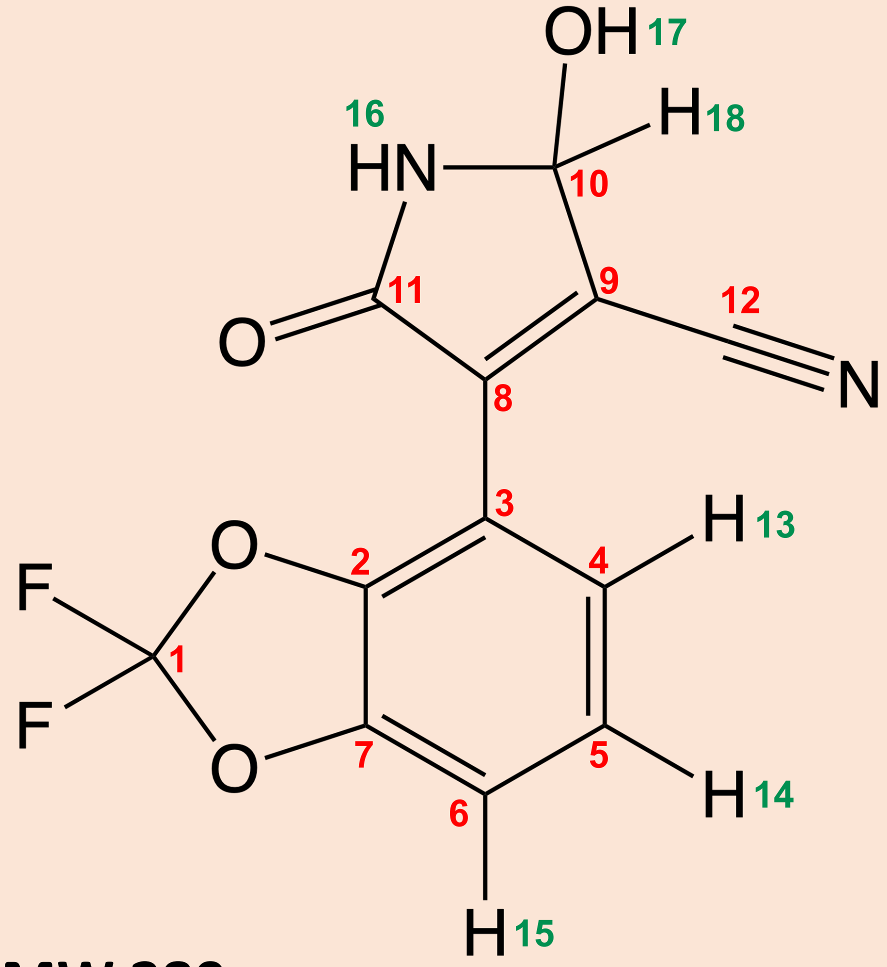

OH
17
H
18
16
HN
10
9
12
O
11
N
8
3
H
13
O
2
4
F
1
F
O
7
5
H
14
6
H
15

MW 280

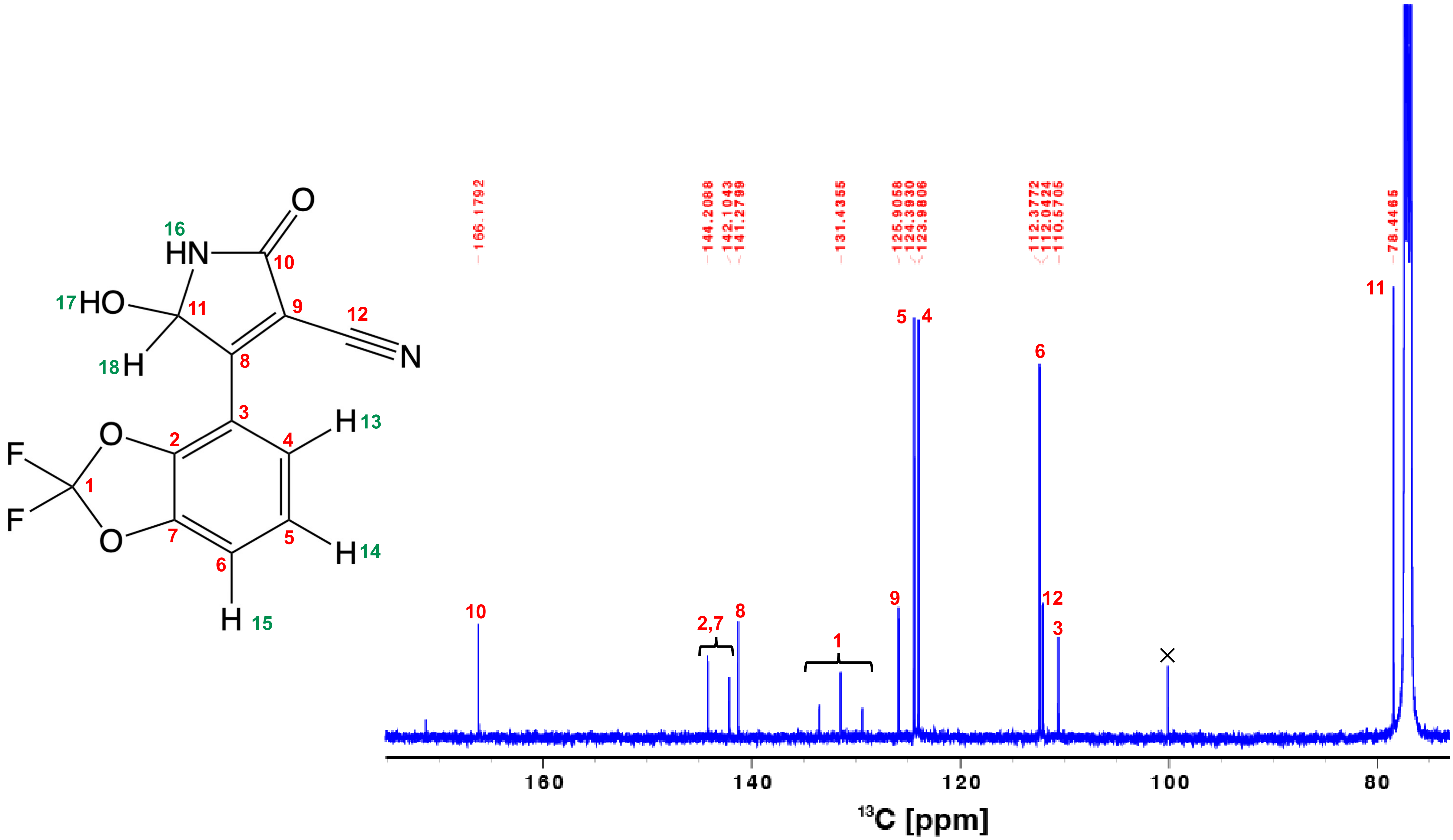

HN
HO
O
N
F
F
O
O
H
H
H
H
1
2
3
4
5
6
7
8
9
10
11
12
13
14
15
16
17
18
166.1792
144.2088
142.1043
141.2799
131.4355
125.9058
124.3930
123.9806
112.3772
112.0424
110.5705
78.4465
10
2,7
8
1
9
5
4
6
12
3
11
160
140
120
100
80
^{13}C [ppm]

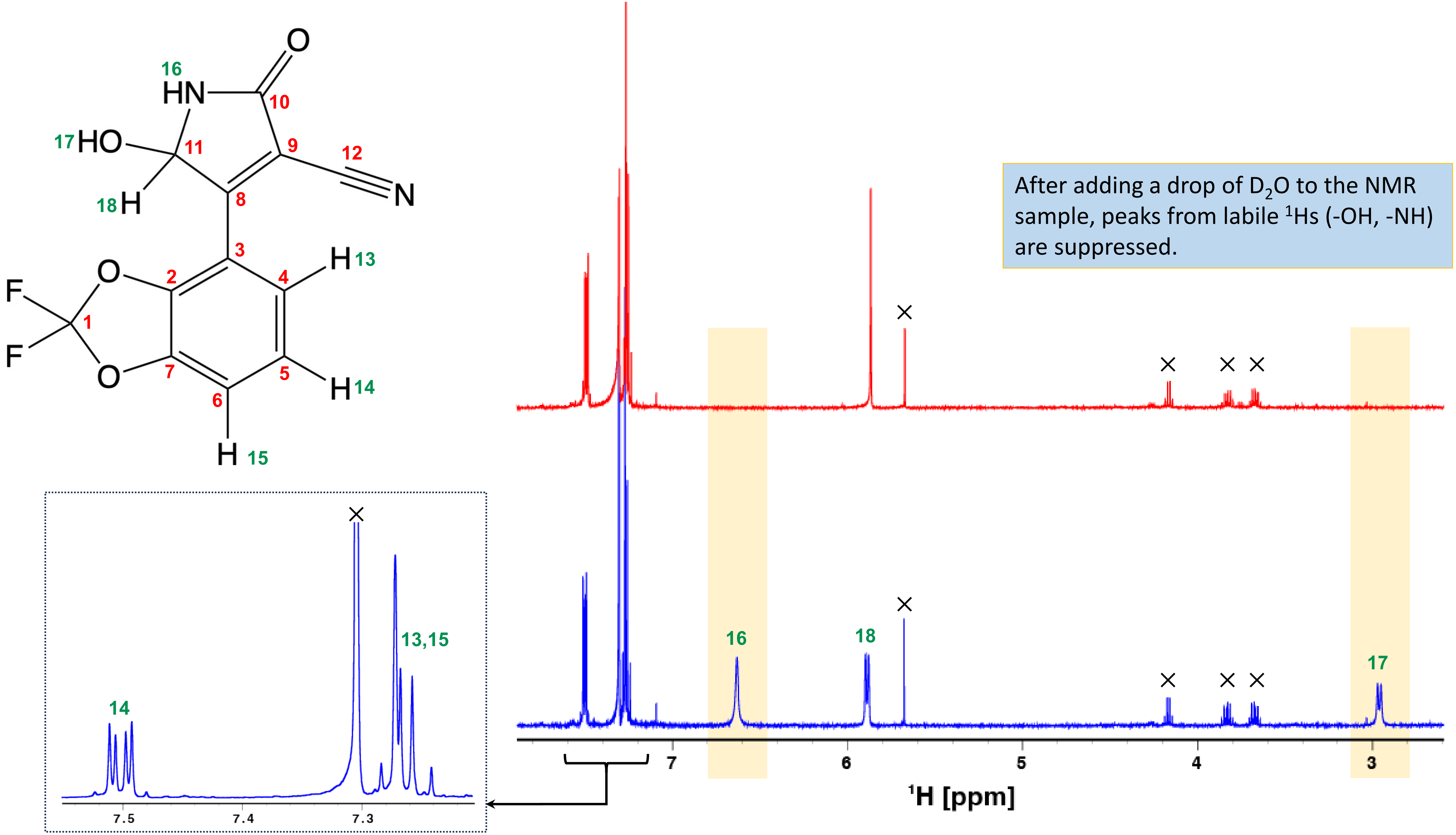

O
HN
16
10
17 HO
11
9
12
N
18 H
8
3
H 13
O
2
4
F
1
F
O
7
5
H 14
6
H 15
After adding a drop of D_2O to the NMR sample, peaks from labile ^{1}Hs (-OH, -NH) are suppressed.
16
18
17
14
13,15
7.5
7.4
7.3
7
6
5
4
3
^{1}H [ppm]

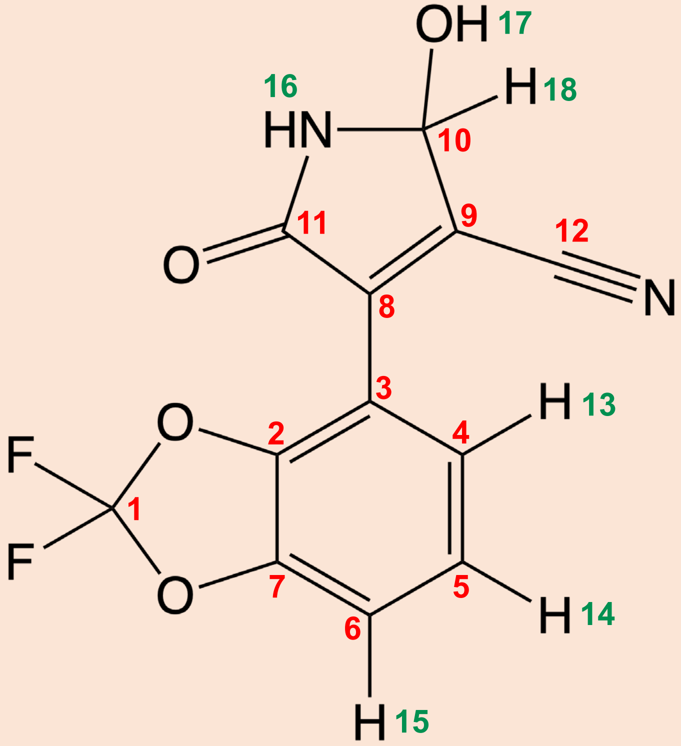

OH 17
16 HN
H 18
10
11
9
12
O
N
8
3
O
H 13
2
4
F
1
F
7
5
O
H 14
6
H 15

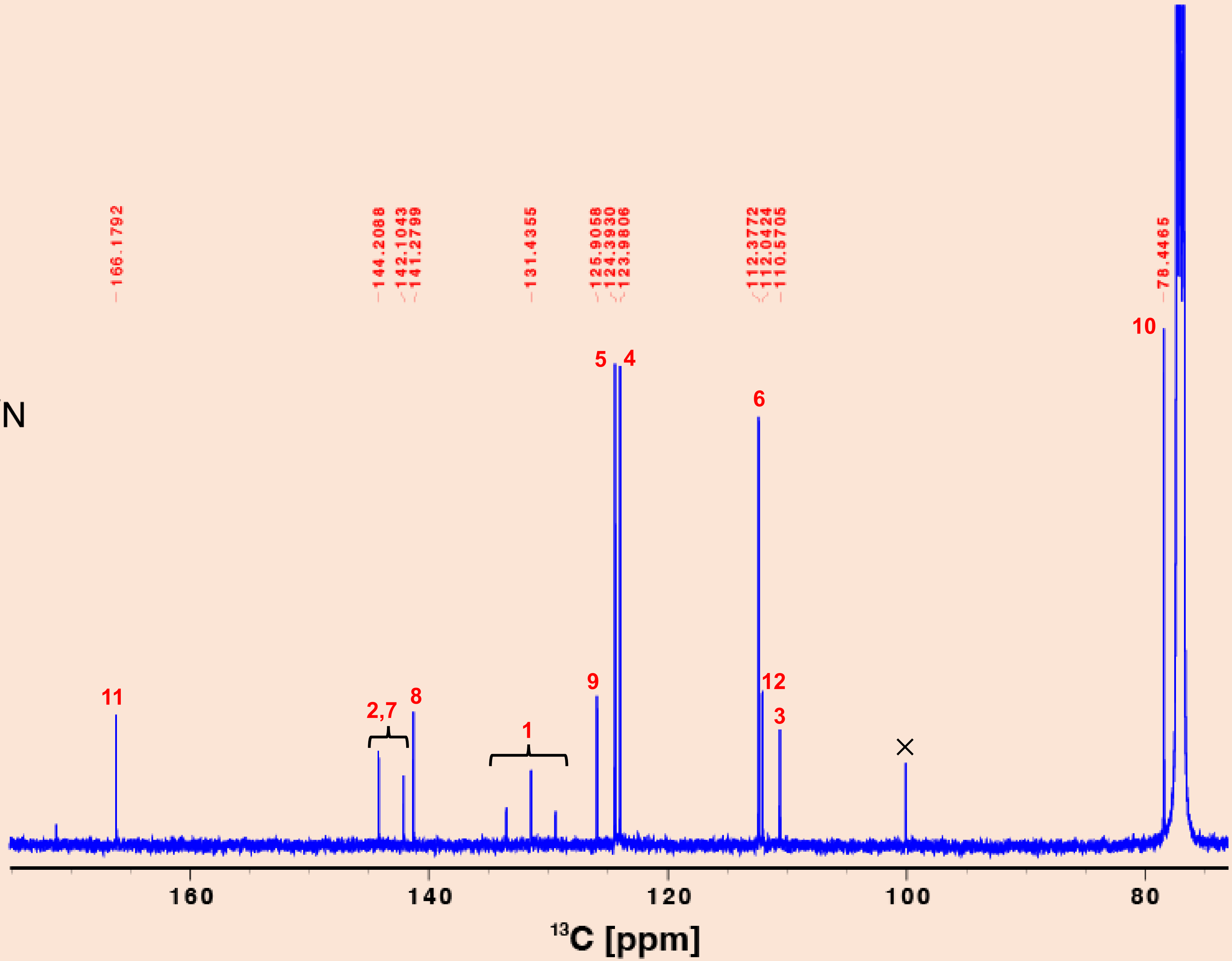

166.1792
144.2088
142.1043
141.2799
131.4355
125.9058
124.3930
123.9806
112.3772
112.0424
110.5705
78.4465
10
5
4
6
11
2,7
8
1
9
12
3
×
160
140
120
100
80
13C [ppm]

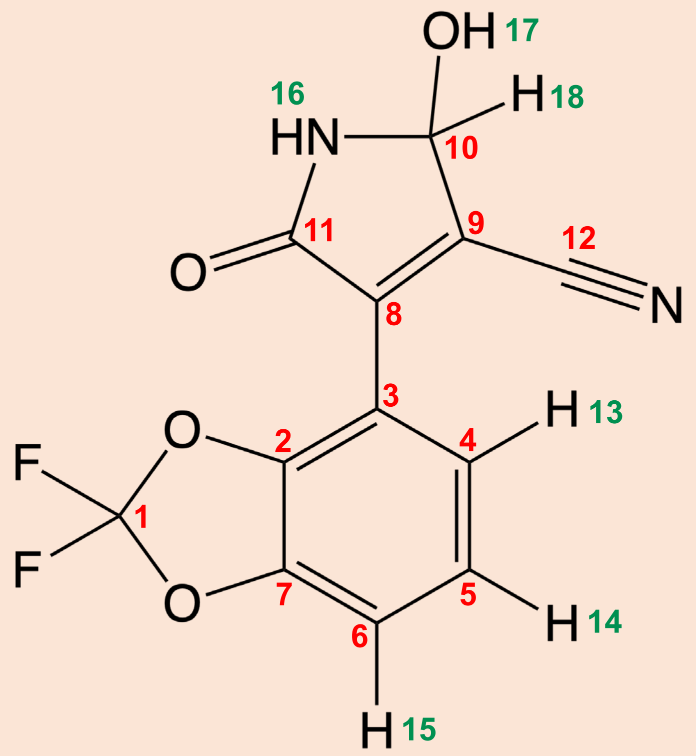

After adding a drop of D_2O to the NMR sample, peaks from labile ^{1}Hs (-OH, -NH) are suppressed.

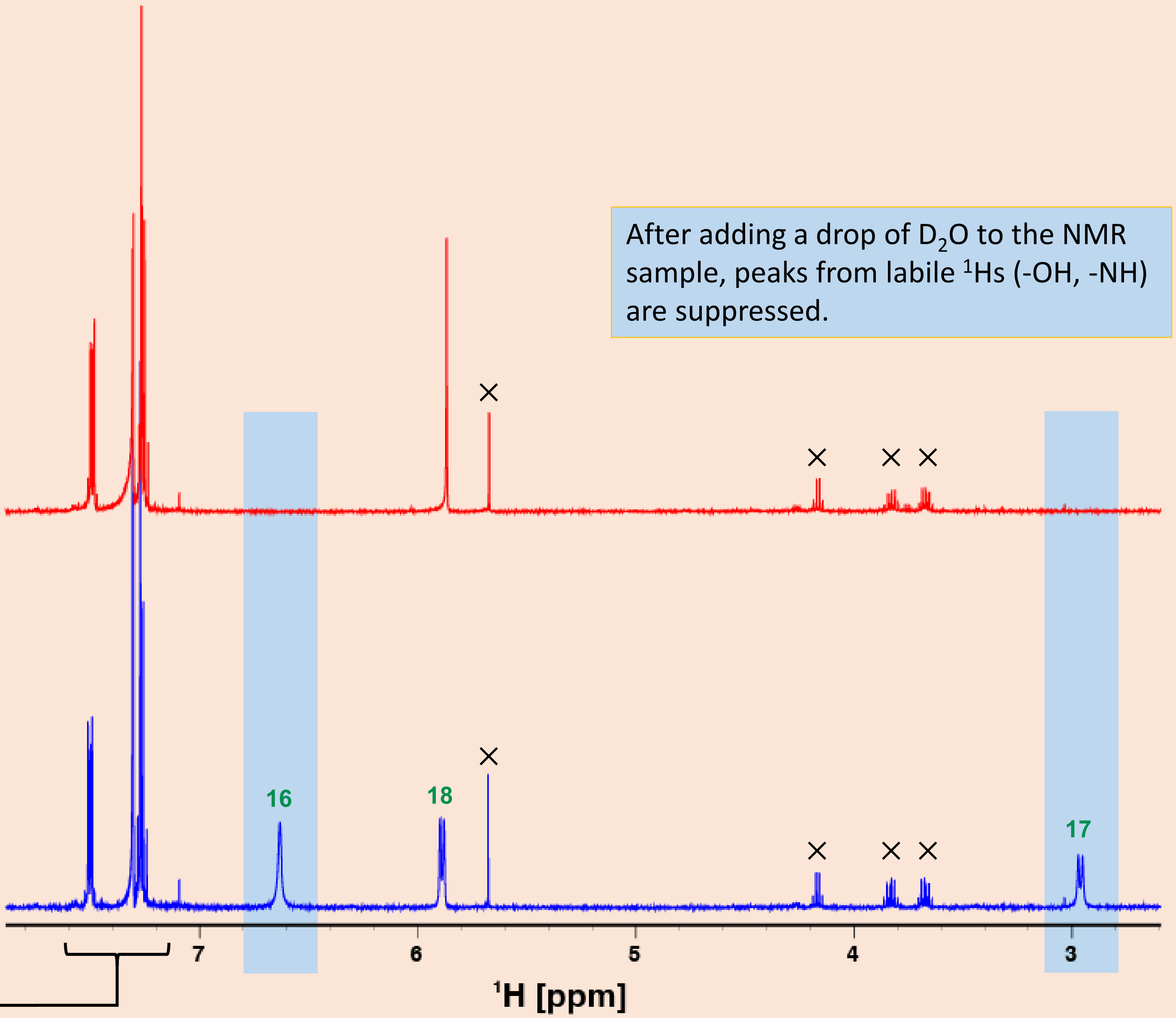

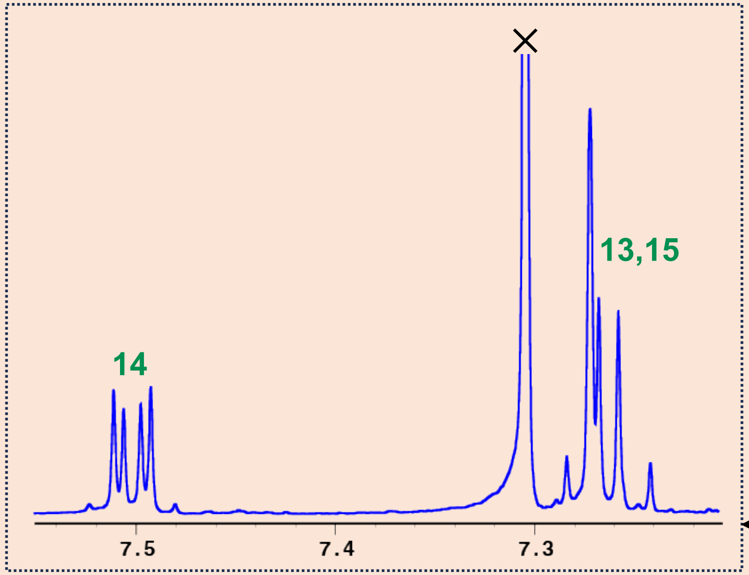

Supplement: Supplemental Information 4 [file peerj-14-21290-s004.pdf]
